# Popular Observations on the Diseases of Literary and Sedentary Persons. To Which Are Added, Hints for Their Prevention and Removal

**Published:** 1820-01-01

**Authors:** 


*Popular Observations on the Diseases of Literary and Sedentary Persons. To which are added, Hints for their Prevention and Removal.* By W. ANDRE PEARKES, Member of the Royal College of Surgeons. Octavo, pp. 144. London, 1819.

IT is with feelings of the deepest regret that we are forced, as one of the Guardians of the Medical Press, to expose the most barefaced plagiarism which we have ever witnessed. The present work is a mere Abstract of Kirkpatrick's translation of Tissot's "Essay on Diseases incident to Literary and Sedentary Persons," and does not contain five pages of original matter from beginning to end! We have taken the pains to compare the two books, page by page; and from alpha to omega we have found but one continued plagiarism, without a single mention of either Tissot or his translator!

But we have something worse than plagiarism to bring against the infatuated author of the work before us, whom, from our hearts we pity, and for whose future good, and not to gratify any unworthy feelings, we point the arrows of severe, yet just censure. Thus at page 19, we have the following passage:

"*I* have occasionally seen men of the first talent, who have done much in the advancement of literature, live, for more than twelve months in complete idotism, and at last die by this species of apoplexy."

Who could imagine that a man, in this enlightened era, would have the courage to personate the illustrious Van Swieten, and usher himself on the stage of medical literature, arrayed in the borrowed *experience* of that distinguished physician! Yet so it is; for even in this *personal* assertion, he has plagiarised the fact! It is a literal version, or rather transcription from page 49 of Kirkpatrick's translation.

"It has given me, [Van Swieten] much concern to see learned men of the first class, who had been very serviceable to literature, live more than a twelvemonth after the loss of their faculties, forget every thing, and at last die of an apoplexy."

Alas, alas, for medical science! Well might Cullen say, that "there are more false *facts* than false *theories* in physic." But we have done. The author has but one course to pursue, for the reparation of the injury which he has committed against the Medical Republic. Let him instantly cancel the Title Page and Preface, and call the book what it really is—"An Abridgement of Dr. J. Kirkpatrick's translation of Tissot's Essay on the Diseases of Literary and Sedentary Persons; *New Edition.*"

The author has somewhat *ominously* dedicated his work to JUSTICE Park.—In performing this most disagreeable part of our duty, we have only obeyed the solemn injunction—"FIAT JUSTITIA."*

